# Foreign

**Published:** 1840-11-21

**Authors:** 


### FOREIGN.

*De Irritide, Commentatio ab Illustrissima Societate Medico Practica quæ Lutetiae Parisiorum floret, in altero certamine die xxvii. M. Septembris, anni* 1836. *Praemio Aureo publice ornata.* Scripsit FRIDERICUS AUGUSTUS AB AMMON, Med. D., &c. Accedunt in Tabb. Aen. ii. Figg. Pictæ xviii. Lipsiæ, 1838, 4to, pp. 48. *Prize Essay on Iritis.* By FREDERIC AUGUSTUS VON AMMON of Dresden, &c. Leipsic, 1838.—Dr. VON AMMON has been for many years distinguished as an able ophthalmologist, and, besides the practice of the profession of oculist, is known as the editor of a journal of ophthalmology, and the author of several able essays on various subjects connected with the anatomy, physiology, and diseases of the eye.

In the year 1834, the Paris Medico-Practical Society proposed as the subject of their prize essay for 1835, the following question: To describe the nature and symptoms of Iritis; to establish the different species of the Disease, and to explain the method of treatment. The essays were to be written in Latin or French, in conformity with the usual rules, and transmitted to M. Alpheus Cazenave, General Secretary to the Society, before the 1st of May, 1835. The present essay, which was received in proper time by the judges, was referred by them to another competition, which was appointed for the year 1836; and in this competition it obtained the prize.

Though the essay received from the commendation of the judges, very unequivocal proof of its merit, the author after receiving it, conceived it to be his duty to revise it carefully, and add, retrench, modify, or rectify, as the subsequent experience of two complete years suggested; and the monograph, accordingly, appears with all those improvements which such revision may be expected to insure.

It was further the wish of the author, that the society should have caused all the figures and delineations with which the essay was accompanied, to have been engraved and published; but this the society declined to do. The author was therefore under the necessity of preparing and publishing these at his own expense; and he has, on this account, been compelled to restrict their number to eighteen. Those who wish a greater variety and extent of illustration, he refers to his Clinical Illustrations of the Diseases and Malforations of the Eyes, Eyelids, and Lacrimal Organs, published in folio at Berlin, in 1838 and 1839.* Dr. Ammon divides his essay into six chapters; the first devoted to the anatomy and physiology of the iris; the second to the nature, symptoms, and cure of the iritis in general; the third to the subject of traumatic iritis and its different forms; the fourth to the subject of serous *iritis* and its differeut species; the fifth to parenchymatous iritis and its various forms; and the sixth to posterior serous iritis or inflammation of the ueva and its effects.

The iris Dr. Ammon justly represents to consist of two thin membranes, an anterior and a posterior, containing between them a celluloso-vascular web, which he terms the proper tissue of the iris, (*tela iridis propria.*) This celluloso-vascular web, he observes, is thin and soft at its external or large circumference, and forming in some parts of the anterior surface, rugæ or folds, various in size, extends to the small or pupillar margin, which is formed by its condensation in a circular form. The orbicular fibres, which many anatomists represent to be found there, Dr Ammon has never observed. The iris, therefore, he concludes, is composed of numerous vessels, nerves, and contractile cellular tissue, forming the continuous circle in the region of the pupil. When this cellular tissue is examined by the microscope, it appears on the pupillar margin abundant, and as if puckered, and the middle substance is thinner than in the ciliary margin.

Dr. Ammon agrees with those who regard the vascular structure of the iris as of the erectile nature, and who ascribe the motions of the organ consequently chiefly, if not solely, to changes in the state of its vascular system. The ciliary arteries, he observes, which form the great and small circles of the iris, are peculiar in their structure, and present a great resemblance to the capillary vessels of the muscles both in external form and course, and in function, which consists in the alternate changes of very rapid expansion and contraction.

The anterior and posterior membranes of this curtain are of the nature of the serous membranes, possessing the same physiological properties, and liable to the same morbid actions. The anterior membrane, or that which forms the membrane of the aqueous humour, appears, when carefully examined, to be villous or velvet-like in structure; a peculiarity which is derived from the presence of minute *villi* or piles on the anterior surface of the membrane, and the very tender vessels of the iris. Its continuity with the membrane lining the cornea is demonstrated in the eye of the fœtus and new-born infant; and the only difference is that the latter is perfectly translucent, and very smooth, while the anterior membrane of the iris is villous and less translucent. This membrane never passes into the posterior chamber of the eye. This anterior membrane is chiefly arterial. The posterior membrane of the iris, which has been long distinguished by the name of *uvea*, is, on the contrary, mostly venous in structure, and secretes *pigentum nigrum.*

The iris is, as it were, enchased, or set into

* Klinische Darstellungen der Krankheiten und Bildungsfehler des Menschlichen Auges, der Angenlider und der Thranenwerkzeuge nach eignen Untersuchungen. Theil i., Berlin, 1838; Theil ii. Berlin 1839, in folio.

the inner margin of the cornea and sclerotic membrane by the ciliary circle or ciliary margin. This part, the minute structure of which Dr. Ammon described particularly in the year 1830, and which he named the *orbiculus capsulo-ciliaris*,* is very important in a pathological point of view.

One circumstance in the anatomy of the organ, very important in explaining some of its inflammatory conditions, we are surprised Dr. V. Ammon does not notice. The iris is doubtless enchased into the cornea and sclerotic membranes of the eye; but its connection with the latter is at once closer and firmer than it is with the former. The ciliary margin of the iris is most extensively connected to the sclerotic coat, and the numerous vessels with which the former is supplied are intimately connected with the latter. Though the sclerotic coat also is itself a colourless membrane, and therefore naturally not very vascular, and its vascularity is then very difficult to be removed. In many cases of iritis, especially when originating in syphilitic or mercurial poisoning, the sclerotic is affected before the iris, and the inflammation extends from the former to the latter; and conversely, when the iris itself is inflamed, it invariably causes considerable affection of the sclerotic, partly directly, partly through the medium of the *orbiculus capsulo-ciliaris*, described by Dr. V. Ammon. This, indeed, is one of the circumstances which renders iritis so difficult to be speedily and completely cured, and so liable to recur.

Iritis, Dr. V. Ammon regards as a disease of the greatest importance, and indeed the cardinal point, as it were, of ophthalmic pathology; for as the iris is situate in the middle of the eyeball, and has an intimate communication with the most important parts of the organ, so the doctrine concerning iritis occupies the central point, as it were, of the whole ophthalmiatric art.

Iritis may be induced by two orders of causes, either internal or external. The latter are such circumstances as wounds or blows on the eyeball. The former consist of those deranged states of the system which are known in this country by the general name of ill health, bad habit, or cachectic conditions, and are designated in the German schools, where the humoral pathology is not and never had been altogether extinct, under the convenient abstract term of *Dyscrasia*, that is, a bad or morbid mixture of the fluids, and which may be represented in English by the analogous term of Distemperature. Whether the hypothetical assumption of *dyscrasia* or distemperature will always account for the occurrence of such diseases as iritis, is not in every case easy to say; nor is it more easy to define this distemperature, or specify the marks by which its presence may be inferred. But it may be sufficient to observe, that there are in general indications of a disposition to inflammation in various textures, more especially chronic; and probably this disposition is induced by the united operation of some morbid poison and cold on the system. Dr. Ammon specifies as instances of the *dyscrasia* or distemperature, a depraved constitution of the blood arising after fevers, and acrimony of the fluids depending upon the syphilic poison, scrofula, the variolus poison, itch, gout, or rheumatism. These forms of distemperature rarely produce iritis spontaneously, he adds; and it requires the concurrent operation of other causes, as exposure to solar heat, or the heat of overheated apartments, wind, smoke, dust, the use of wine, seasons of the year, hard labour, and especially exertion of the eyes, all of which easily excite the disease in cachetic subjects.

We are surprised that in this enumeration the author does not mention as a cause of iritis, a state of the system which is by far the most frequently observed in this country to be favourable to the production of the disease. The great majority of cases of iritis take place in the persons of those whose systems have been more or less impregnated with mercury, in some instances to a very large amount. The charging of the system with this mineral is then the distemperature or *dyscrasia* which disposes to the disease; and in general so strong is the disposition thus induced, that a very slight exciting cause, as exposure to cold, or a little too much use of the eyes, is quite sufficient to induce the disease. We are aware that it is the custom to ascribe cases of iritis taking place under such circumstances, to the operation of the syphilitic poison, uncontrolled, and not expelled from the system, and that the appearance of the symptoms of iritis is then regarded as the signal for the renewed exhibition of mercury. But, independent of the fact that it is very doubtful, and assuredly not ascertained, that the syphilitic poison even by itself produces iritis, it is a fact which can be shown to repose on the foundation of numerical results, that iritis is infinitely more common after the use of mercury, than after any other of the causes already specified.

We must here take the liberty of noticing and rectifying another fallacy in this matter. Iritis is said by almost all authors, and Dr. Ammon is among the number, to follow rheumatism or the rheumatic *dyscrasia*. Now, though we do not say that iritis never accompanies rheumatism, or even that it rarely accompanies or follows that disorder, we must state it as the result of observation somewhat extended, that the disorder termed rheumatism, which it usually and most commonly accompanies, is

* Der *Orbiculus Capsulo-ciliaris* eine Verbindung welche in menschlichen auge zwischen der hintern Flache der Ciliar-fortsatze und der vordern Linsen Kapsel wand besteht Vom Herausgeber. Zeitschrift fur die Ophthalmologie von Prof. Dr. F. A. V. Ammon. Dresden, 1830.

not genuine rheumatism, but periosteal inflammation, which, from its site, and from the circumstance of the pains being most intense during the night, is always regarded as chronic rheumatism. If, however, in such cases, the affected parts be examined, it is found that the periosteum is thickened, rough, irregular, and generally very painful. These periosteal inflammations are very generally excited and maintained by mercury; and the great disposing cause both to these and also to the iritic attacks, is, doubtless, according to what we have observed, the phlogistic state of the system, and its extreme susceptibility to the detrimental operation of cold, induced by the operation of mercury.

Dr. Ammon is at a loss to determine whether the acute cutaneous disorders have any influence on the production of iritis; and he observes, that, as the acute exanthemata most commonly attack the skin and mucous membranes from the commencement, and seldom affect the serous membranes towards the close of the disease, he thinks that these disorders should not be regarded as mere affections of the skin, but as inflammatory or sympathetic irritations. He allows that this is less applicable to the serous membranes, and especially to that of the iris; for if towards the close of typhous fever, small-pox, scarlitina, or measles, this membrane is affected, this affection terminates either in albuminous or in puriform exudation, and not, as in exanthematous diseases, in desquamation. It is a matter of fact that iritis takes place after typhous fever, after small-pox, after scarlet fever, and after measles. These attacks of iritis, however, he argues, occurring at the close of such diseases, are to be regarded as metastatic, if by metastasis be meant the migration of a disease from one organ to another, while the primary disorder subsides on the migration. But inflammation of the iris also, he thinks, is truly dyscratic, that is, depending on distemperature; because it takes place in the system after its fluids have been depraved by fever or other disorders.

To these views we feel it impossible altogether to assent; and we think that English physicians and oculists will not be astonished that we do not. We are much inclined to call in doubt the fact of metastasis in cases of the kind mentioned by the author, and to consider the whole of them as the natural effect of the unresisted or aggravated course of the disease.

The iritis which is observed to follow typhous fever is not so much mere inflammation of the iris, as a part of inflammation of the eyeball at large, and especially the sclerotic coat, or of the cornea, or of the choroid coat. It is most usually observed to follow or accompany one or other of these affections, and is never observed to take place by itself, unless in persons who have been under mercurial influence at the time at which the febrile disorder was developed, or after it had subsided.

The variolous iritis comes on in a mode a little different. Almost invariably it is the result of one or two variolous pustules on the cornea, giving rise to inflammation and ulceration of the entire thickness of that membrane. In this state the inflammatory action extends through the cornea, and if near the insertion of the large or outer major of the iris, it gives rise to inflammation in the latter membrane. In other instances it causes inflammation of the iris merely by the extension of the morbid action to the membrane of the aqueous humour. In a third class of cases, the variolous pustule proceeding to ulceration, the cornea is perforated, the aqueous humour escapes, the air is admitted; the iris, either with or without being pushed to the aperture in the cornea, becomes the seat of inflammation; and the whole eyeball is involved in the same destroying process, which terminates in general confusion of all its textures, in staphyloma and atrophy of the ball.

Of the iritis which succeeds scarlet fever we are happy to say that we have little or no experience.

The iritic inflammation which takes place after measles is not very different from that which follows small-pox. The morbillous or rubeolous action always affects the ophthalmic mucous membrane; and if the inflammation be intense or considerable, it passes to the cornea and the sclerotic coat, and thence to the iris. It may happen that this ophthalmic inflammation may be confined to the ophthalmic mucous membrane; it may happen that it may subside in the general disorder, and, above all, the disorder of the facial and bronchial mucous membrane subsides; it may happen that, after continuing for a few days in the congestive and irritative state, it may subside without having so materially distended the vessels that they easily empty themselves, and without giving rise to any chronic inflammation or any inflammatory product. But we know that the rubeolous inflammation does leave both the palpebral and the ocular mucous membrane in a state of chonic inflammation, exactly as it leaves the bronchial and pulmonic vesicular membrane in that state; and it cannot be wonderful if this inflammation should pass to the cornea or the sclerotic coat, and thus affect the iris. In all this we perceive nothing like metastasis. We recognize only the extension of inflammatory action from one texture to another, and the natural persistence of inflammation, or its lingering in various textures, if it has not been moderated or subdued by the prompt and seasonable employment of remedies.

The gouty or athritic iritis is the only one which can be said to be indicative of a generally disordered state of the system. But that general disorder, it must be observed, is part of the general state of distension and plethora of the vascular system, and is both created and aggravated by full living and the abdominal plethora to which persons who indulge in the pleasures of the table are liable. It may be doubted whether there is any other *dyscrasia* than this; and it is a clear proof of the nature of this *dyscrasia*, that the disease is always moderated and abated, and sometimes wholly removed by diminishing the fulness of living, and restraining the diet. "Iritis," says the present author, "is not unfrequently the last link in a long chain of many evil symptoms; especially among the gouty advanced in age; among the strumous, and those who are affected with inveterate syphilis; and the same takes place in those in whom the abdominal viscera are obstructed, or in whom the portal vein is the source of disorder."

In this class of persons, we would observe, the affection of the sclerotic coat previous to, or along with, that of the iris is conspicuous. Not only does the iris display the change in colour indicative of inflammation in the case of light and contracted pupil; but round the cornea is seen the deep pink-coloured ring, which indicates the chronic inflammation of the sclerotic coat, while the patient feels the soreness, fulness, and painful tension all around the eyeballs.

Inflammation of the iris may either arise in, and be confined to, the iris itself, or it may arise like inflammation in other textures of the eye, and hence pass to the iris, or it may pass from the iris to the other textures. In the first case it is primary or idiopathic; in the second, it is secondary or symptomatic; and in the third, it is complicated. Inflammation may also attack the whole or part of the iris, or may affect the membrane previously sound, or more or less diseased. This gives rise to the distinction into universal or partial, simple or complicated. Again, the disease may be seated in the anterior surface, in the posterior surface, or in the substance of the membrane. The two former constitute different forms of serous iritic inflammation; the latter is the parenchymatous iritic inflammation. Lastly, all these different forms may be united.

Dr. Ammon further thinks that iritis may be distinguished into several stages, three at least; the first, that of congestion or inflammatory irritation of the iris; the second, true inflammation; the third, more violent inflammation; and as to rapidity of course he makes it to be either acute or chronic. It is also when it has once taken place, very liable to recur; and it is then very likely to destroy the membrane, or even the eye. When it takes place in those blind of one eye, it is much more likely to prove pernicious and destroy the organ, than where the other is sound, or where both are previously sound.

The symptoms of iritis are arranged by the author in two classes; the objective symptoms, and the subjective symptoms.

The objective symptoms are those which are distinguished by surgeons as the sensible signs. They consist in the change of colour in the iris, changes in its brilliancy and position, changes in the state of the aqueous humour, morbid exudations on the anterior or the posterior surface of the membrane, and in its surface, changes in the state of the pupil and in the motions of the iris, swelling in the venous circle, a peculiar sort of redness in the conjunctiva, and a greater or less degree of blepharitis, that is, inflammation of the palpebræ. Though these may be mentioned as the pathognomonic objective symptoms of iritis, yet they might be greatly increased in number, if those symptoms are regarded which attend complicated iritis, that is, if along with the iris, the ciliary circle, the ciliary processes, the capsule of the lens, and the *corona ciliaris*, or if the choroid coat, retina, sclerotic coat, or cornea be attacked with inflammation.

The subjective symptoms of iritis consist of those dynamic changes or disorders in the functions of the eye which attend or follow iritis, viz. intolerance of light, pain in the iris, and various changes in vision.

Dr. Von Ammon then considers these symptoms separately and in detail; and we shall notice those which are most important.

One of the most characteristic symptoms is change in the colour of the iris. The natural colour is always more or less changed; for instance a bluish iris assumes in the inflammatory state a greenish colour, and the deep-seated colour becomes reddish. Besides these changes in colour, which also loses its lustre, the natural brilliancy of the membrane is impaired or disappears, and the characteristic structure of the membrane, as connected with its brilliancy, is disturbed or altogether destroyed. These changes, which are easily recognized by comparing the sound with the inflamed eye, begin at the pupillary margin of the iris, and pass gradually to its ciliary margin. These changes in colour and brilliancy are ascribed to the unusual accumulation of blood in the vessels and parenchyma of the iris, and the change in the state of the blood itself, whence the secretion of that matter to which the iris owes its clearness, its colour, and its brilliancy, is either diminished or entirely changed. It is allowed that the individual globules of this fluid change both their shape and colour in inflamed parts.

Besides the change in colour, the site of the iris is also changed. It loses its funnel-like shape at the recess of the pupil, and advances nearer the posterior wall of the cornea. This change is also caused by the excessive accumulation of blood in the vessels of the membrane, causing it to be distended and swelled, and accordingly, as it cannot easily swell backwards, it does so in the forward direction. This shows that it is not so much the iris that changes its position as the distended parenchyma of that membrane, by which its central portion is protruded forward. This must be distinguished from strabismus, which the protruded iris often simulates.

In the first stage of iritis the aqueous humour becomes muddy—a change which depends on the derangement in the secretive function of its membrane. It is also not unfrequently more copious than natural.

The pigment or colouring matter of the iris is changed in inflammation; for it is secreted sparingly, abundantly, or morbidly. If the quantity secreted be less than natural, paleness in the colour ensues, and great derangement in vision. If the quantity of pigment is increased, in general the colours become brown and indistinct; the shape and aspect of the iris also may be so changed that protuberances are formed in it, various in colour and shape. Lastly, if the pigment itself be changed, the colour of the iris is also changed or destroyed. (*Iridallochrosis.*) The author has observed so large a quantity of yellow pigment in the middle of the circle of the iris, that the latter being elevated assumed a stellated arrangement, which seemed as it were a crown placed on the pupil. He has also often observed, especially in chestnut coloured eyes, so large a secretion of pigment over the whole web of the iris, that its anterior surface appeared sprinkled with a large quantity of brilliant powder.

The morbid secretions furnished by the inflamed iris consist either of a jelly-like mass, or albumen, or puriform fluid, or blood.

The exudation of lymph, which is at first fluid and jelly-like, is always fraught with danger to the eye, in so far as it very often produces adhesions, destroys the mobility of the iris, fills up the pupil, and consequently destroys vision. Dr. V. Ammon distinguishes two forms of lymphy exudation, one jelly-like and semifluid, not necessarily causing adhesion, indeed, rarely causing adhesion, and the other plastic lymph, or that which does cause adhesion. It is almost needless, however, to say, that these two are mere varieties of the same morbid effusion.

The forms in which these exudations may be deposited are too various to be enumerated in this place. It is sufficient to say, that they may cause every variety of irregular pupil;—the circular, the oval, the double elliptical segment, like the pupil of the cat, the triangular, the quadrilateral, the trapezoidal, and the irregular polygonal. When they connect the pupil to the lens, the effect of these adhesions is to form various species of *synechia* or closure of the pupil. Even short of all this mischief, inflammation may render the inner margin of the iris thick and puckered, and incapable of motion; and very often this result is accomplished without the effusion of much lymph, or with the effusion of a small quantity into the interstitial cells of the membrane.

The secretion of lymph on the *uvea* or posterior surface of the iris always almost constitutes posterior *synechia*, either partial or total, according to the quantity of lymph effused, either in separate filaments, or in large quantity. The former state is known by dilating the pupil artificially, when the form is regular; the latter, by the immobility of the thickened iris and the subsequent occurrence of cataract. In some instances, the exudation extends towards the ciliary processes and the choroid coat, filling the pupil with a new morbid mass.

The exudation of lymph into the substance of the iris, which has been already partially noticed, gives rise to great changes in the aspect, colour, and shape of that membrane. Its living, movable, and erectile aspect disappears, and it seems like a hard, dead, membrane in the eye. Its colours, also, are greatly changed—yellow-whitish and black mixed, or reddish and brown. The surface of such an iris always appears thickened and irregular. It is still more irregular, when, as oftimes happens, the lymph has assumed the form of drops or little tubercles. This state of the iris Dr. Ammon at one time denominated *Iridoncosis* (*tumescentia iridis*;) but he now calls it, *Iridauxesis* (*ampliatio vel incrementum iridis*;) reserving the former name to designate abscess of the iris.

The termination of iritis in suppuration, Dr. V. Ammon allows occasionally to take place. He thinks, however, it is most common after wounds of the iris and cornea, and after violent shocks of the eyeball. Sometimes, also, it takes place in gouty subjects; but he allows that it rarely depends on iritis alone, and is more frequently a sequela of partial or general *ophthalmitis.* This puriform exudation assumes the form of hypopyon, that is, a collection of purulent matter in the anterior chamber, or what is more rare, it forms an abscess of the iris, which bursts and pours its contents into the anterior chamber. The secretion of purulent matter in *iridopyosis*, according to Dr. V. Ammon, takes place suddenly, and not uncommonly when the disease appears to abate; and the author generally observed it towards night, which was spent without sleep from increase of pain. The purulent matter does not from the first assume the appearance of hypopyon; suppuration begins in separate flocks towards the inner and lower part of the iris. These are dissolved in the aqueous humour, which is thus rendered turbid; and as the secretion of matter proceeds, this fills the bottom of the anterior chamber, and forms hypopyon. He thinks that few physicians are aware of the marks of incipient hypopyon, which, he says, are only to be known by accurate examination of the inflamed iris by the aid of the lens. Hypopyon may be accompanied or followed by ulceration of the cornea, and sometimes by onyx. This state is most dangerous, as by it the cornea is often ruptured, or eroded, or ulcerated, so that the humours escape, and staphyloma following, the patient is the prey of incurable blindness.

The second kind of suppuration in the course of iritis consists in the formation of abscess in the parenchymatous substance of the iris, forming the iridoncosis of Dr. Von Ammon. This is observed in partial iritis, sometimes in the pupillary margin, sometimes in the ciliary margin; but it always takes place near the *vasa vorticosa*, and is wont to be of a yellow colour. The tumour varies in size, being sometimes flat, sometimes prominent; a circumstance which may lead the observer to mistake it for an excrescence. Such tumour of the iris (*iridoncos*) is seldom absorbed. More frequently it is ruptured, and the matter is effused into the anterior chamber of the eye, forming secondary hypopyon. The walls of the opened abscess immediately after the rupture float to and fro like flocculi, in the anterior chamber, and gradually by absorption and contraction disappear. In the place of the abscess albuminous matter, yellow, black, or white may appear, and form an anterior synechia; or a black spot, or a yellow-white spot, or a yellow spot is left in the iris. When these spots are black, they readily imitate the appearance of the iris; and the author is persuaded that those observers who maintain that, after abscess of the iris, a new pupil may arise, and say that they have seen it, confound this sort of black spot with the pupil. He further thinks that in the place of abscess of the iris calcareous excrescences may be formed; but he allows that on this point he requires further experience.

Dr. V. Ammon has not observed ulcers in the serous part of the iris, excepting those which follow the rupture of an abscess; and which he justly remarks, are not entitled to the name of ulcers, since abscesses of the iris opened in this manner are speedily healed by the effusion of coagulable lymph. He allows, however, that it is worth while to direct attention to this point, in order that some precise information on the subject of iridelcosis or ulceration of the iris may be obtained. Analogy is against the position, as the serous membranes are not liable to primary ulceration. The author does not, however, deny the possibility of the occurrence of ulceration in the iris, if it has been destroyed by suppuration, and if the abscess is open, and not disposed to unite.

On the anterior surface of the inflamed iris are seen, especially by the aid of a lens, vessels loaded with blood, various in size, course, and colour, according to their seats, and according to their origin.

Blood-vessels become manifest in serous iritis, when it becomes chronic, and when the serous membrane of the iris is affected with hypertrophy. These come into view at the ciliary margin, and more towards the pupillary margin, where they disappear in such a manner, that it is difficult to ascertain whether they go to the uvea or sink into the substance of the iris. From these vessels blood may be effused, causing discolouration of the iris, black spots on it, or muddiness of the aqueous humour; but more frequently they furnish the lymph, which assumes various shapes and colours, and at length they disappear.

In parenchymatous iritis, the author has frequently seen a beautiful net-work of blood-vessels in the very beginning of the disease, usually in the middle of the iris, or towards its ciliary margin. These vessels, which are manifestly in the parenchyma, and therefore not easily distinguished, are not new, but the proper vessels of the membrane distended and filled with blood. They are visible only a short time; and when exuditaon commences they become indistinct and disappear.

He allows, however, that towards the close of the parenchymatous iritis, when it has proceeded to *iridauxesis*, there arise in the anterior surface of the degenerated iris new blood-vessels, which run tortuously between the morbid elevations of the iris, are of a blue or red-black colour, and not unfrequently are so much distended with blood as to cause hæmorrhage, (*hæmopthalmos*,) either by diapedesis or diabrosis. Such varicose vessels usually form a circle, which comes into view towards the pupillary margin, and from which issue individual branches, proceeding through the pupil into the posterior chamber.

This exudation of blood in iritis may take place in a small or great amount, or may be pure, or mixed with lymph or purulent matter. The effusion of blood in iritis, whether from new or from distended vessels, is always an indication that the economy of the iris is very much deranged.

Blood thus effused is, however, very often absorbed, especially if the iris was sound immediately before the attack of inflammation. When, on the other hand, the substance of the iris has suffered several attacks of inflammation, when the nature of the iris is dyscratic, that is, we suppose, when its circulation is previously in an unhealthy state, when inflammation, originally acute, often recurs, the blood is seldom absorbed, but assumes a brown or black colour, causing black spots on it, contributes to the formation of *iridauxesis*. Blood is sometimes effused into the substance of the iris, causing *hypouema*, which is known by the blood separating into its two parts, the clot and serum, as it does when drawn from a vein.

Lastly, either all or several of these morbid exudations may be combined in the same case.

The changes in the pupil have been partly noticed as the effects of inflammatory thickening, induration, or exudation and adhesion. The margin or border of the pupil is often more indistinct than in the natural state, and so tinged with *pigmentum nigrum*, that it appears to be denticulated or serrated;—an appearance which is not caused by loss of substance, but by the profuse secretion of this black matter.

The pupillary margin, however, when carefully examined, is irregular, especially in inflammatory irritation of the iris, or chronic iritis, and in bluish-gray eyes. (*To be continued.*)

*Practical Observations on the Diseases of Peru, described as they occur on the Coast and in the Sierra.* By Archibald Smith, M. D.

I.—Diseases of the Coast continued.

*Gastric and Intestinal Hæmorrhage.*—The climate of Lima, and of the coast of Peru generally, is observed to induce among the inhabitants of those regions, a peculiarly weak and lax state of the gastric and intestinal tissues, as well as of the capillary vessels connected with them, which subjects these parts to frequent, though rarely fatal, sanguineous discharges consequent on various causes of local irritation.

The following examples may serve to show how such disorders usually appear; and are commonly connected with deranged digestion, though not always with organic disease.

*Ex.* 1.—A little girl, three or four years old, of Spanish parentage, and fair delicate skin, being in her usual health, was put into the cold bath; felt chilled in it; was soon taken out, and shortly after voided one copious stool of dark blood. Next day she was affected with prickly heat; and without further trouble, or the use of medicine, her bowels, as now indicated by healthy alvine motions, recovered their natural condition.

*Ex.* 2.—A brown or Zamba slave girl, about ten years of age, had no appetite, and felt dull and heavy for several days. The lady whose property she was gave her a dose of manna, which did not operate much, if indeed at all, as a purgative. But soon after it was taken the girl vomited some blood; and then, as if by a charm, her spirits returned, and she was quite well.

*Ex.* 3.—A military officer, worn down by arduous service, and the want of regular rest, was attacked with tertian. His bowels having been previously relieved in some degree by emollient ptisans and clysters, according to the fashion of the female attendants of the country, the physician who was called recommended a simple infusion of bark, alternated at the proper medicinal hours with low diet. This treatment was attended with vomiting of blood and discharge of blood from the bowels; the blood voided by stool being dark in appearance, more or less clotted, and very fetid. I first saw the patient when in this state, and ordered that the bark should be discontinued. He was now put on a thin arrow-root diet, with linseed and mallow-water for ordinary drink. The belly was anointed with oil and covered with flannel, with a view to keep up an equable circulation, and a starch enema was administered once a-day. Under this treatment, the tertian was very slight at the next corresponding period; and after it had terminated, the usual gentle purgative of the Limenian practitioners was resorted to; namely, a dose of manna dissolved in tepid whey. By this means the bowels were opened freely, and the stools assumed a natural appearance. All the bad symptoms now disappeared; and in two or three days more, under good nursing and attention to regimen, he was perfectly convalescent.

*Ex.* 4.—An elderly lady, who enjoyed general good health while she only eat what she knew would agree with her, was sure to be seized with discharge of blood from the bowels as soon as she happened to eat any thing that she could not easily digest, or what too much irritated the duodenal or general intestinal mucous membrane; thus illustrating the old observation, *ubi stimulus, ibi fluxus.*

This sort of case is not of rare occurrence among the people of Lima; but those who are occasionally affected in this manner commonly recover from the ailment without the aid of the physician. They habitually resort to such simple domestic remedies as an emollient enema, anointing the belly with almond oil, and drinking liberally of a ptisan, which is composed of mallow, linseed, and rose water, with the addition of a little sugar or any simple syrup.

*Ex.* 5.—A stout, active, and middle-aged bronzed woman, sat down to breakfast in her usual good health, and eat of boiled Indian corn half ripe and in ear, (*choclo,*) *tamal*, or pork baked with bruised maize, (usually enveloped in a piece of plaintain leaf,) *camotes*, or sweet potatoes, several hard eggs with fresh curd, and a fair allowance of chocolate, besides. An hour after this, as she conceived, most simple repast, she washed her body with cold water, which soon induced chills and shivering, with a general feeling of indisposition and pain in the belly. These symptoms were followed by a feeling of constriction and irritation at the arms, and there now came on evacuations of pure blood mixed with bile. Under these circumstances she resolved upon an act of self-denial, viz., to eat no more that day. She diluted what she had eat at breakfast, by repeatedly, in the course of the day, drinking freely of linseed water sweetened with syrup of roses; and the result was, that on the following morning she rose perfectly well.

*Ex.* 6.—A gentleman, well advanced in life, and long affected with chronic hepatitis, was directed to take a dose of manna in oil, the favourite purgative *orchata* of the old doctors of Lima; and on the night of the same day on which he took this savoury potion, he was seized with hæmatemesis. A physician was instantly sent for, who, finding that ice, a popular remedy in such cases, was not at hand, prescribed an anodyne draught with twenty-five drops of laudanum. This dose the stomach rejected, and therefore the same quantity of the opiate was again repeated, and he passed the remainder of the night quietly.

Next morning a consultation of two native physicians was called. They determined that the patient should be bled* in the forenoon; and

* A bleeding in Lima seldom exceeds twelve or in the evening he was put into the warm bath by their direction. The unfortunate gentleman was no sooner taken out of the bath, which had probably induced a fatal internal hæmorrhage, than he became as cold as the grave, and expired.

*Ex.* 7.—A young Englishman, who had been for some time a visitor in one of the great commercial houses in Lima, was seized with discharges of blood from the bowels. The case became one of consultation; and I was one of the physicians consulted. We learned from the account of his former medical attendant, Mr. Kidstone of Lima, that the young gentleman's attacked had been of hepatic origin, but found from his actual state that ulceration had probably taken place in the alimentary canal. Hence vessels of considerable size appeared to have been laid open; for the blood which was passed by stool, from being dark and grumous, became florid and arterialized in appearance.

After many profuse and clotted discharges of blood, repeated from day to day, the intestinal hæmorrhage at length became still more alarming. Exhaustion with faintishness ensued; and the consequence was that the hæmorrhage naturally ceased. Now the patient confined himself rigidly to milk alone, as his only drink and nutriment. There was no longer any appearance of blood in the stools, and convalescence went on uninterruptedly. While on the milk diet he daily evacuated large pieces of curd, which, however, did not seem to occasion irritation in the bowels. He embarked for Europe, and arrived safely in England.

*Ex.* 8.—A clever woman, entrusted with the management of the commercial affairs of her husband, is habitually troubled with a hæmorrhoidal discharge, and frequently exposed to many sources of mental annoyance. Though a person of a cheerful and most charitable disposition, yet such is the prevaling influence of the depressing emotions over the digestive functions in her case, that she never experiences molestation in mind without its being attended, or quickly followed, by corresponding bodily indisposition.

Having on one occasion been irritated, and put out of temper, and consequently seized with an attack of colic, attended with much flatus in the bowels, I was sent for. She felt great drowsiness and heaviness abut the head, with a tendency to fall into a state of insensibility.

After the attack had thus commenced, the patient had intervals of respite; but the disease recurred in fits attended with severe colic pain; strong chattering of the teeth; general convulsive motions, and particular contortions of the face. These paroxysms did not continue long at a time, but were followed by short intervals of quietude and stillness, though not of insensibility.

As she recovered from these fits, under the administration of blood-letting and opium, incredible quantities of dark coagulated blood were voided *per anum*. Uneasiness was felt at the region of the liver, and colic pains attended the sanguineous stools. The mildest laxatives and emollient enemata were resorted to; and sinapisms or blisters were applied to the abdomen, without in any degree removing the intestinal local irritation and pains, or the existing hæmorrhagic discharges.

Ice, the usual remedy, and iced acidulated drinks, failed to afford that relief which at length was happily derived from pills of opium combined with a small portion of acetate of lead. This efficacious remedy speedily checked the inordinate intestinal hæmorrhages, in which there was often no appearance of fæcal matter. After a few days' rest, the patient was removed to Chorillos, in a very weak and reduced state; but she made a very favourable recovery.

A year or two after this attack, she had another equally severe, in all respects, excepting the intestinal hæmorrhage, which was merely hæmorrhoidal and moderate in quantity. But on this latter occasion, the liver was unusually sensible when pressed upon, as was also the spleen, though in a less degree, and, indeed, the whole abdomen was somawhat tender and impatient of pressure.

A dose of castor oil with twenty drops of laudanum, aided by emollient clysters, served to quiet irritation and freely open the bowels; by which means the attack, with all its lethargic and convulsive symptoms, was removed. She was now put under a mild mercurial course, which, by correcting hepatic disorder, afforded great relief from the habitual hæmorrhoidal affection, and likewise from colic uneasiness, and frequent *xaquecas*, or periodical headachs, to which she had long been subject, and which were always sure to come on after any painful mental excitement.

I may remark, that for the *xaquecas* or headachs to which this lady was subject, *ayudas* or clysters, and iced drinks were her almost daily and familiar remedies, when in any degree threatened by these molestations. In most cases these means, with a thin farinaceous diet, and occasional warm-baths, afforded her a temporary respite. This line of treatment was not peculiar, but is that usually resorted to by the Limenese, in the generality of the *xaquecas* to which I formerly alluded as symptomatic of disorders of the digestive organs.

I have known, however, iced beverages not to accord with persons addicted to drink much ardent spirits, when affected by intestinal irritation or hæmorrhage.

I should not fail to mention in this place that hæmorrhoids are so common in Lima, even

---

sixteen ounces when ordered by native practitioners. They are fond of smaller bleedings, frequently repeated, in the common inflammatory disorders of the country.

among those who present to our observation no palpable visceral obstructions, that as an ailment, they are little thought of until from costiveness, cold, long rides, or over-exertion, &c. they become inflamed. While they do not cause much uneasiness, persons habitually troubled with them think little of voiding blood with their stools; and this blood, they vulgarly imagine, descends from the scapulary region. They call it *sangre de espaldas*, a kind of generic name, not confined to the discharge from piles, but applied, as well, to other more formidable discharges of blood from the intestines.

I have met with innumerable instances where this sort of sanguineous drain was habitual, and sometimes copious, without producing any appreciable weakening effects, or injuring, upon the whole, the general health.

We sometimes find the heart very strongly, though only sympathetically, affected in cases of intestinal hæmorrhage, of which I would relate the following as a striking instance. A middle aged gentleman, of a full habit of body and pale countenance, was subject for several years to frequent as well as abundant discharges of blood from the bowels. He was an ardent politician, and easily agitated by the operation of mental emotions. When walking in the street, a feeling of approaching syncope, but without actual fainting, was readily induced, even in his ordinary state of health; and when the vessels happened to be overloaded, and the pulse was full,—as in those states of local congestion that immediately preceded the periodical discharges of blood from the bowels, any mental disquiet or excitement was sufficient to occasion the peculiar feeling of oppression called *fatiga*, and a violent palpitation of the heart, attended by pain shooting across to the left and sometimes into the right arm; thus simulating, or realizing a paroxysm of *angina pectoris*.

This gentleman had been often bled for these attacks, but experience showed that bleeding could only afford temporary relief; for the disorder was probably sustained by a general plethora. The local congestion and consequent intestinal hæmorrhage appeared to originate from the peculiar influence of disagreeable emotions or strong passions, in causing accumulations of blood in the abdominal vessels. Low diet, open bowels, and living in the country, removed from scenes of political intrigue, and great occasions of mental excitement, were chiefly the means that afforded this patient some alleviation under so protracted and distressing a disease of which I know not the final result.

The following case, though foreign to the general subject of gastric or intestinal hæmorrhage, it may be worth while to introduce here, as not only manifesting the influence of the mind over the action of the intestines, but also on the uterine system.

A remarkably stout, active, and ruddy complexioned Limena, who had borne several stout boys, and owed much of her unusually robust constitution to her ordinary occupation of superintending a dairy, was one day on a visit in a house, where the conversation started happened to be disagreeable to her; the consequence was, that she almost immediately felt a call to stool, and had to retire. This took place at an early hour after dinner, when the food was still crude and undigested in the stomach, and, therefore, she drank a glass of water, and, by way of precaution, betook herself to bed.

When she awoke next day, she felt something suddenly give way within her, and then there came a strong gush of blood *per vaginam*, which she partly stopped by drinking iced-water, and applying to the loins cloths dipped in vinegar and cold water. She was at this time in a pregnant state; but, notwithstanding, after this sudden attack of hæmorrhage, she experienced several irregularities in what she considered to be the catamenial discharge; and being also apprehensive lest she should have suffered some serious damage inwardly, she consulted a physician regarding the nature of her case, and the propriety of sea-bathing. He told her that, if she were rash enough to try the sea-bathing, the inevitable consequence would be abortion. The patient, however, followed her own counsel. She went to the sea-port of Callao; and her health and appetite were improved by the bracing effects of the sea-bathing.

*Empacho*. (*Impactio*, Lat. Constipation.—Under the title of *empacho*, I do not mean to describe any new disease peculiar to Peru; but I here use this term, which admits of a double acceptation, in preference to costiveness, with a view to familiarize my readers with a native expression in constant use; and referring to an assemblage of symptoms which, in some shape or other, is vulgarly supposed to be connected with almost every form and modification of gastric or intestinal disorder, to which the inhabitants of Lima more especially are subject.

The word *empacho*, then, is generally used in two distinct senses, which require to be explained.

In the one instance, it is employed to denote an unnatural collection of alvine sordes, or an excessive accumulation of fecal matter in the bowels; in the other case, it signifies a casual lodgement in some part of the alimentary canal of any undigestible matter, such as the coriaceous portion of a fig, grape, or date, keeping up irritation, and probably inducing diarrhœa. The former example is in vulgar language called simply *empacho*; the latter is *empacho pegado*, or adherent *empacho*.

It is only of the first of these affections that I mean to offer some account in this place. But for the popular prejudices of the Limenese connected with their notions on the subject of the *empacho*, I may refer to what I have written elsewhere regarding this and other vulgar prejudices.*

A torpid state of the bowels, though not at all so prevalent, (except in the aged,) as its opposite extreme, diarrhœa, is frequent and troublesome; but is not always reckoned a disease among the sedentary portion of the Limenians, who often require the help of the *Xeringa* or clyster, as an indispensable means of relief.

We can imagine that a costive habit may be acquired by eating much more than is necessary for nutriment or easy digestion; and in Lima those who do not always consult solidity in quality, often make up for it in quantity. When the tone of the intestines is once diminished, the habit of costiveness is easily formed; and each renewed distension of the colon and rectum renders them more disposed to become the passive receptacles of undue fecal deposit. I have seen the rectum so distended even in a child, by an immense accumulation of hard and impacted feces, as to have lost its natural power of contraction, when no agent less active than an enema with croton oil was required to procure relief.

Such a statement as the following is sometimes heard from a person of a costive habit. "It is only at the expiration of many days, and with much trouble, and the help of clysters, that I am relieved in my bowels. When after a succession of days eating but not evacuating, the intestines become thoroughly stuffed, (*atorados*,) I feel no longer any appetite for food; and then come colic and vomiting, and I am not relieved until the belly be freely opened." This state constitutes what is called by the native physicians *a stercoraceous empacho*, (Lat. *Impactio*; obstruction;) and the patient has probably been so long inured to stimulate the rectum by clysters, that he finds the great intestines, though distended like a sausage, no longer obedient to these ordinary assistants; and, therefore, he comes to the doctor for a purgative which gives temporary ease; but when once freed from the troublesome burden by the active operation of castor oil or some such remedy, the person goes away and eats as before, until the bowels are again so well stuffed, that no more can be eaten till the bowels have been emptied by the renewed employment of the remedy.

It is not unusual to hear complaints concerning the stercoraceous repletion or empacho in old persons, from which, at the end of four or five days, perhaps they are partially relieved by voiding with uneasiness a hard scanty motion; and twice or thrice in every month, one so molested may have a day of looseness in the bowels or a kind of salutary flux, consisting of hardened or perhaps scybalous feces, mixed with a portion of liquid discharge, and is consequently left at rest for several days after.

I visited an old Indian troubled with this sort of habitual costiveness and occasional diarrhœa, in whom the heart was affected and the liver vastly enlarged; and when the fecal accumulation was not timeously removed by physic or clysters, he used to complain of "una fatiga de la muerte"—a death-like oppression about the stomach and heart. And I observed in the same individual in whom the occasional returns of diarrhœa were preceded by external coldness of the extremities and entire body, that the preternatural palpitation at the heart went on increasing for months, until at length it became violently fluttering, when all the great arterial trunks in the thoracic cavity seemed to have shared in the same struggle and irregular action before death,—in which the complicated infirmity of the old man, after many alternate alleviations and aggravations of symptoms, at length ended.

The following case, as it occurred in his own person, was one day related to me by an aged Lima practitioner, who consulted me on the best way of treating his ailments.

"For many months I have been ailing, and they treated my case as an aneurism, with ice and vinegar, &c., because they found that in the abdomen between the stomach and navel, a great elevation or tumor with strong palpitation had been formed. But observing that I neither perspired, expectorated, nor exonerated the belly, I resolved upon taking a dose of manna in whey, by which I voided very large and long detained feces, and thus the elevation or tumour decreased. But I fear that, with all the ice and vinegar I was made to use for the supposed aneurism, some of my viscera have become obstructed. The tepid baths only relax my bowels so as to fill them with air, that has no exit downwards, and moves about in the bowels, and so distends them, that there is no relief without a purgative. When I do not take the manna I have no passage in my bowels; and when the belly is bound, the tumour supposed to have been aneurismal, becomes quite elevated; with a rumbling of air in the intestines, and attended with flatulent eructations, and a jumping motion—'*salto*'—or palpitation, which always ceases when the bowels are evacuated. So you see, sir, they were all mistaken who said that my disease was aneurism."

In this instance, the tumour, which had long continued stationary in the transverse arch of the colon, where it was moved by the strong palpitation underneath, until dislodged by the manna, gave rise to the erroneous notion of an internal aneurism. Such a mistake I have known several practitioners to have fallen into on other occasions as well as this; and the distension of the colon, as happened in the above case, from fecal detention and flatus, I have witnessed in other instances attended with *pyrexia*, and confounded with acute *hepatitis* and enlarged liver, till a dose of salts and senna, or some other suitable purgative, dissipated the tumour, and with it the physician's delusion.

* See Peru as it is, Vol. i. p. 56.

Strong but not constant palpitation of the abdominal aorta and its chief branches, is a common sympathetic affection, and I have known it arise in a man whose general health was good, in consequence of eating a bit of chicken, before the slight irritation or excitement induced in the *primæ viæ* by an ordinary dose of a purgative medicine, had entirely subsided.

---

*Longevity in Russia.*—In the government of Kasan there were, among the old people who died in 1839, 5 of 100, 7 of 101, 3 of 102, 3 of 103, 2 of 104, 3 of 105, 1 of 107, 2 of 108, 1 of 112, and 2 of 115 years old. In the government of Woronesch, there were 33 of 100, 11 of 105, 3 of 110, 3 of 115, and 2 of 120.—*Brit. and For. Med. Rev., from Zeitschrift für die gesämmte Medicin.* Juin, 1840.

---

*Mortality of St. Petersburg.*—An evident proof of the unhealthiness of the climate and mode of living of St. Petersburg is supplied by the extracts from the church books respecting the numbers born and dead in the foreign religious communities in the city, in the course of the year 1839. The Greek church, Mahomedan, and Armenian congregations are excepted.

*Ibid.*, *from Ibid.*

* The Finlanders come in great numbers to be apprenticed at the various manufactories, and very frequently die of phthisis about the time of puberty.

*Danish Medical Statistics.*—In the *Danish Medical Library* for January, February, and March, of the present year, there are copious extracts from the Transactions of the Royal Society of Health for 1839. The first is an account of the prevailing diseases in Denmark, with the exception of Laaland and Falster, during the year 1838. These are small-pox, scarlet fever, measles, catarrhal complaints, typhus, which has been very prevalent, rheumatic fever, puerperal fever, psora, syphilis, croup and hooping-cough, which last, after an absence of sixty years, has re-appeared among persons of all ages in the Faroe Isles. We are also informed that in the same year (1838) 18,813 persons were vaccinated in the whole kingdom for the first time, and 1052 were re-vaccinated. That in Iceland 481 were vaccinated for the first time, and 647 were re-vaccinated.

The tables of births and deaths are not very precise. The number of accidental deaths and suicides, strangely enough united together, was 514.—*Ibid.*, *from Bibliothek for Laeger.* Jan., Feb., March, 1840.

---

THOUGHTS ON MEDICINE.

See the critic take his measure and his balance; he measures you by his height, weighs you by his weight, and estimates you by his value; he has no other rule for his judgment. If, in writing, you show energy and fire, he says it is mere emphasis and declamation; if you display elegance he calls it tinsel and affectation; if you are simple and natural, he asserts that you fall into common-place; if you show learning, according to him it is pedantry; if you are sparing of quotations you are ignorant of what has been done before you. The magnifying glass of his malevolent criticism alters and distorts every thing. What is to be done, then? Wait. This great man, so difficult to be satisfied, takes up his pen, and, wonderful to say! his subject is hacknied, his principles false, his logic pitiful. Add, that his style is incorrect, heavy, flat, and wearisome, and no one is ignorant that the style is the man.

---

A new idea can have no permanence, if passion, enthusiasm, and strong self-love, do not assist it; they are powerful and active springs; the difficulty is to bring them into the service of the good, the useful, and the true.

---

*General rule.*—When the reviewer forgets the work, to attack the author, and to penetrate the sanctuary of private life, he ceases to be a journalist, and becomes a libeller.

---

How many diseases, pains, dangers, and infirmities, men would avoid, if they were fully imbued with that great principle of hygiene and of wisdom, that pleasure sometimes enters into the composition of happiness, but never forms its essence.

